# Preoperative Ultrasound-guided Core Biopsy of Axillary Nodes for Staging of Clinically Negative Axilla in Breast Cancer Patients – A Pilot Study

**DOI:** 10.7759/cureus.6718

**Published:** 2020-01-21

**Authors:** Shaista Afzal, Imrana Masroor, Asma Munir, Romana Idress, Poonum Khan, Shaista Khan

**Affiliations:** 1 Radiology, Aga Khan University Hospital, Karachi, PAK; 2 Breast Surgery, Aga Khan University Hospital, Karachi, PAK; 3 Histopathology, Aga Khan University Hospital, Karachi, PAK

**Keywords:** core biopsy, breast cancer, axillary lymph node, sentinel lymph node biopsy

## Abstract

Objective

The aim of the current study is to determine the feasibility and accuracy of ultrasound-guided core biopsy for staging the axilla in clinically node-negative patients with invasive breast cancer.

Introduction

Historically, in breast cancer patients, axillary lymph node dissection was performed to stage axilla. Because of the high morbidity of axillary lymph node dissection, sentinel lymph node biopsy (SLNB) became the standard of care in patients with clinically node-negative breast cancer. However, SLNB is expensive, time consuming, can cause morbidity and can be complicated by seroma formation, sensory nerve injury, lymphedema, etc. Many centers rely on the availability of frozen section on sentinel lymph nodes to avoid a second procedure with the accuracy of procedure ranging from 73 to 96%, however, the availability of frozen section is limited in our part of the world. Pre-operative identification of axillary node positivity in patients with clinically negative nodes by ultrasound imaging of the axilla would allow one-stage axillary clearance and can decrease the need for SLNB from 21% to 70%. The aim of the present study is to determine the accuracy and feasibility of ultrasound-guided core biopsy to stage the axilla in clinically node-negative breast cancer patients, comparing with final histopathology as gold standard.

Material & methods

This was a non-randomized, prospective interventional study, done at Radiology Department of Aga Khan University Hospital. All patients diagnosed with breast cancer (histologically proven) with clinically negative axilla and ipsilateral positive axillary ultrasound were included. These patients underwent axillary lymph node core biopsy. If the result was negative they were subjected to SLNB. Histopathology result was taken as gold standard.

Results

The sensitivity of ultrasound-guided core biopsy was 88%, specificity 100%, positive predictive values (PPV) 100%, negative predictive values (NPV) 89.28%, diagnostic accuracy 94%.

Conclusion

In conclusion, the present study demonstrated high accuracy of ultrasound-guided axillary lymph node core biopsy in breast cancer patients with clinically node-negative axilla. Positive core biopsy results can thus obviate the need for sentinel lymph node biopsy and allow breast surgeons to directly proceed to axillary lymph node dissection.

## Introduction

In patients with breast cancer, axillary lymph node status is an important factor that not only provides prognostic information, but also determines the medical and surgical management options [1,2]. Over the years there has been a transition from more radical procedures to conservative approach. Same holds true for the staging and management of axilla. Historically, axillary lymph node staging was performed by means of axillary lymph node dissection (ALND). Because of the high morbidity of this procedure, sentinel lymph node biopsy (SLNB) became the standard of care in patients with clinically node-negative breast cancer [3]. However, SLNB also has some morbidity and anesthesia risk. Not only is it expensive and time consuming, SLNB can be complicated by seroma formation, sensory nerve injury, lymphedema and limitation of the range of shoulder motion [4]. Furthermore, in order to avoid a second procedure many centers rely on the availability of frozen section analysis of the node. In our part of the world not all the centers have the availability of frozen section analysis. Moreover, the analysis of published data shows that the accuracy of frozen section analysis with a combination of Haematoxylin and Eosin (H&E) staining and immunohistochemistry on sentinel lymph nodes is between 73 to 96% [5]. The presence of micro metastasis, lobular histology, and lymphovascular invasion has been found to be the independent predictors of false negative results on frozen section. This means that patients who are identified with a node-positive disease by permanent section may require completion of ALND, hence requiring a second procedure, increasing the surgical morbidity. These clinical scenarios represent 40% to 50% of patients treated for breast cancer [6].

Pre-operative identification of axillary node positivity in patients with clinically non-palpable nodes/negative axilla would allow one-stage axillary clearance, avoiding the step of SLNB. As clinical examination is unreliable in determining node positivity, pre-operative diagnosis presently depends on imaging of the axilla using imaging modalities such as scintimammography, ultrasound (US), computed tomography, magnetic resonance imaging, and positron emission tomography.

Since the early 1980s, many authors suggested axillary ultrasound to detect axillary lymph node metastases in women with breast cancer. However, the accuracy of US alone seemed to be too low [7]. In 1990s, ultrasound-guided fine needle aspiration or core biopsy of sonographically suspicious lymph nodes was shown to increase the specificity of nodal staging [8-12]. Later on different studies have reported a role for image-guided staging of the axilla preoperatively and the node positive patients can then undergo directly to axillary clearance, avoiding the SLNB. This results in better selection of patients for sentinel lymph node biopsy, i.e., those patients who do not have disease in the axillary nodes on image-guided sampling. Pre-operative staging of suspicious lymph nodes detected by US-guided core biopsy can decrease the need for SLNB from 21% to 70% [13,14].

The overall sensitivity of US-guided biopsy for the evaluation of axillary lymph nodes in the initial staging of breast cancer ranges from 58% to 91% and specificity from 97% to 100%. The false-negative rate for needle core biopsy ranges from 8% to 12% [15-17].

The aim of the current study is to determine the accuracy and feasibility of ultrasound-guided core biopsy to stage the axilla in clinically node-negative breast cancer patients, with final histopathology after SLNB or axillary node sampling/dissection as gold standard.

## Materials and methods

**Figure 1:**
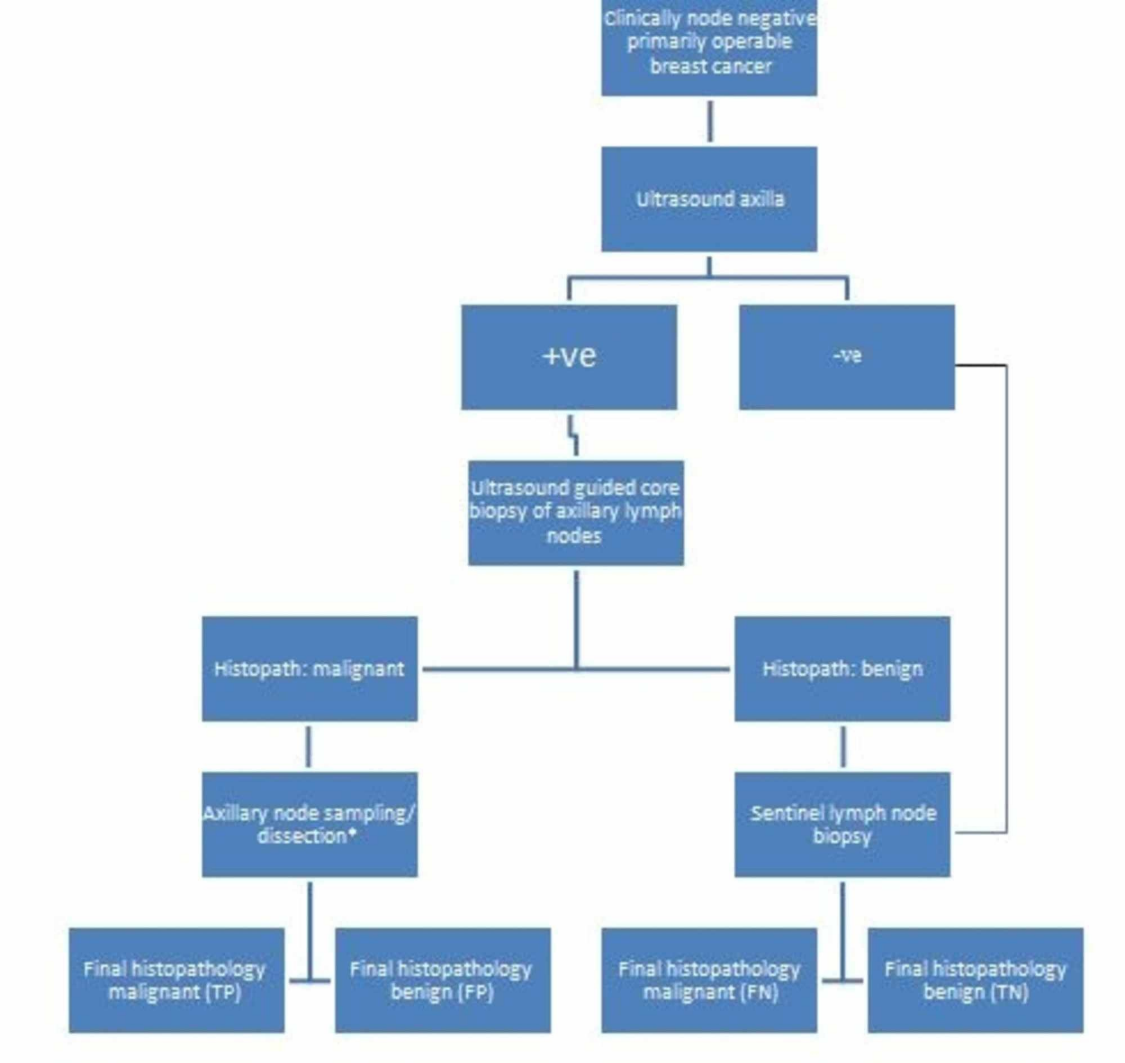
Flow chart showing patient selection. * Dictated by the size of the tumor.

This was a non-randomized, prospective interventional study, conducted at the Radiology Department of Aga Khan University Hospital from June 2015 to June 2017. The inclusion criteria were females with histologically diagnosed breast cancer who had clinically negative axilla and on axillary ultrasound lymph nodes showed (1) eccentric cortical enlargement (>3 mm) or lobulation with displacement of hilum, (2) absent hilum or irregular borders, (3) hypoechoic echo texture, (4) spherical node, and (5) perinodal vascularity [13-15]. These nodes will be labeled as suspicious lymph nodes and will be included in the study. Patients with known breast cancer with palpable axillary nodes, patients planned for neo-adjuvant chemotherapy and patients who were pregnant or lactating were excluded. All patients with suspected positive axillary lymph node on ultrasound underwent ultrasound-guided core biopsy of most abnormal lymph node. Patients with known nodal disease on core biopsy were planned for axillary node sampling or dissection as governed by primary tumor size. Patients with negative core biopsy were subjected to SLNB. Patients with normal axillary ultrasound were also subjected to SLNB (Figure 1). Approval was taken from hospital Ethics Review Committee and informed consent was obtained from all patients. A sample size of 625 was calculated using NCSS PASS 11 (NCSS, LLC, Kaysville, UT), to achieve 81% power to detect sensitivity of 85%. The target significance level was set at 0.05. A pilot study was conducted with 50 patients to validate the technique in our setup. Data was collected and analyzed using SPSS 22 (IBM Corp., Armonk, NY). Sensitivity, specificity, positive and negative predictive values were calculated. A grant of 2037 USD (Rupees 326,000) was approved by University Research Council for these 50 patients. The study was also presented at the 7th World Congress on Breast Cancer, 2018 [18].

## Results

**Figure 2:**
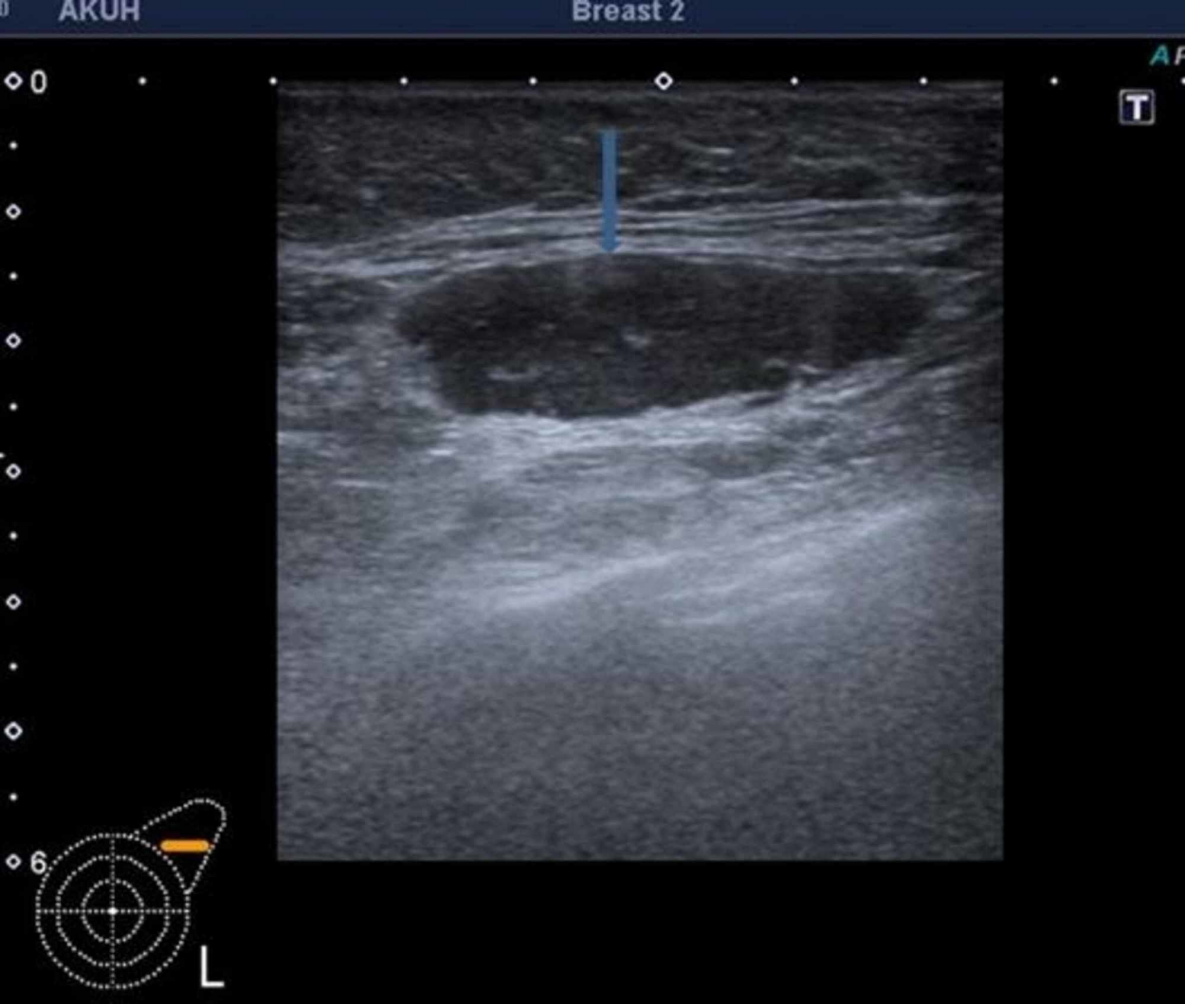
Ultrasound showing an enlarged left axillary node with loss of fatty hilum and thickened cortex (arrow).

The mean age of patients was 51.16 years with SD ± 12.61 (Minimum 30 and maximum 80 years). Out of the 50 patients, the biopsy-proven malignant lesions were equally distributed in both breasts with 25 lesions on each side. The maximum number of lesions were in upper outer quadrant that is 60% (n = 30), 18% (n = 9) in lower outer quadrant, 12% (n = 6) and 10% (n = 5) in upper inner and lower inner quadrants. Majority of primary lesions exhibited all signs of malignancy on ultrasound with 74% (n = 37) being hypoechoic, with irregular and microlobulated margins and increased vascularity. The commonest feature of suspicious lymph nodes on ultrasound was increased cortical thickness of more than 3 mm (Figure 2), seen in 90% (n = 45). Absence of fatty hilum in lymph node was seen in only 6% (n = 3). Majority of patients, 84% (n = 42), had infiltrating ductal carcinoma grade 2 and 3. The number of true positives was 22; true negatives were 25, with three false negatives cases. There was no false positive case in our study. The sensitivity of ultrasound-guided core biopsy was 88% and specificity was 100%. The positive and negative predictive values were 100% and 89.28%, respectively, and the diagnostic accuracy was 94%.

## Discussion

Locoregional lymph node involvement is a crucial component in the staging of patients with invasive breast cancer. Lymph node status represents a major prognostic factor and can influence adjuvant treatment, such as radiotherapy, intraoperative accelerated partial breast radiotherapy, immediate reconstruction and axillary surgery in case of neoadjuvant chemotherapy. SLNB has supplanted ALND as a gold standard for the operative nodal staging of T1-2 N0 breast cancer. Although SLNB is less invasive and associated with lower morbidity than ALND, up to 50% of sentinel nodes will contain tumor deposits and require complete ALND [19]. Imaging for axillary node staging of women with primary breast cancer has developed over time and women should now be triaged to initial SLNB or ALND according to most accurate preoperative diagnostic information [20]. Our study shows that axillary ultrasound and core needle biopsy of the suspicious axillary lymph nodes have adequate sensitivity and high specificity and is a useful test to triage patients regarding the methods to address the axilla surgically. A systematic review done by Alvarez et al. reported sensitivity ranging from 54.7 to 92.3%, and specificity ranging from 80.4 to 97.1% [21]. The sensitivity reported in our study is also within the above range although we performed core biopsy of only those lymph nodes that showed suspicious features at ultrasound. On the other hand, some studies have done biopsy of lymph nodes if they were seen on ultrasound, either with normal or abnormal morphology [22, 23]. The specificity can be explained by the fact that we did core biopsy using an 18 gauge needle, whereas Jankowski et al. did both fine needle aspiration cytology (FNAC) and core biopsy of lymph nodes. The false negative rate was 12%; this is low but still indicates that there is room for improvement in the field of preoperative assessment of the axillae and emphasizes that ultrasound ± biopsy cannot be totally relied upon to stage the axillae [24]. Consequently a negative or inadequate result of ultrasound ± biopsy, can help triage patient to the precise form of axillary surgery, i.e., sentinel lymph node dissection (SLND) [24]. The fact that ultrasound is operator dependent may have implications for the widespread application of this technique. The false negatives are linked to the size of the metastasis. Needle biopsy only samples a portion of a lymph node and therefore may miss clusters of tumor cells present elsewhere in the lymph node. Similar to the study by Garcia-Ortega et al., no patient with micro metastasis was detected in the present study [25]. The false positive rate is an important performance measure for a test that provides staging information. We did not have any false positive patients; however, this could be related to small sample size.

Gurleyik et al. reported the diagnostic accuracy of ultrasound-guided FNAC for the evaluation of suspicious axillary lymph nodes in patients with diagnosis of breast cancer [20]. Their statistical measures of performance are lower than the values noted in the present study where core biopsy was used to sample suspicious lymph node as shown in Table 1.

**Table 1:** Comparison of statistical measures of the performance of FNAC and core biopsy in patients with positive axillary US. FNAC: Fine needle aspiration cytology; US: Ultrasound; PPV: Positive predictive values; NPV: Negative predictive values.

	Sensitivity	Specificity	Accuracy	PPV	NPV
Gurleyik et al. [20], US and FNAC	69.6	100	81.6	100	68.1
Khan et al. [18], US and Core biopsy	88	100	94	100	89.2

In a study by Bhandari et al., the authors compared the diagnostic accuracy of the two techniques of axillary lymph node sampling and reported higher accuracy of core biopsy (96.2%) in comparison to FNAC (90.8%). Their study also demonstrated that in terms of simplicity and safety, the two procedures were parallel [26].

Another study by Ahn et al. comparing the sensitivity of two procedures, i.e. FNAC and core biopsy for staging axilla in breast cancer, reports high sensitivity of both procedures and recommends FNAC on account of low cost and being minimally invasive [27]. In our setup, there is no provision of cytopathology service in the radiology department for immediate evaluation of FNAC slides and these have to be sent to laboratory for verification of the adequacy of the sample which increases the procedure time. In addition, in our setup the charges of core biopsy of axillary lymph node, which are done in the same sitting along the biopsy of the breast lesion, have been subsidized as package deal (i.e., 34 USD), which in comparison to FNAC (i.e., 63 USD) is lower. Hence no extra burden is imposed on patients and they are offered a test with better accuracy and comparable safety.

Ultrasound of the axilla, with core needle biopsy of the suspicious lymph nodes improves selection of breast cancer patients for SLNB or ALND with a higher accuracy than triage based on clinical and ultrasound evaluation alone. This is a benefit to the patient in terms of morbidity as it avoids unnecessary ALND. However, the study also reinforces evidence that ultrasound, even with core biopsy, does not have a 100% negative predictive value to forgo operative axillary staging based on current standard of care.

The small sample size and the possible variability among the radiologist who performed the axillary ultrasound and core biopsy of axillary lymph node are the limitations of our study.

The major focus of the study was to determine the feasibility and accuracy of ultrasound-guided core biopsy, i.e., tissue sampling technique, for which consecutive breast cancer patients were included and this possibly introduced selection bias.

## Conclusions

In conclusion, the present study demonstrated high accuracy of ultrasound-guided axillary lymph node core biopsy in breast cancer patients with clinically node-negative axilla. The result of these biopsies thus determines the type and extent of surgical procedure to address the axilla, i.e., patients with positive core biopsy results can be spared from sentinel lymph node biopsy, allowing breast surgeons to directly proceed to axillary lymph node dissection.
